# Microbiological Quality and Safety of Fresh Pork Meat with Special Reference to Methicillin-Resistant *S. aureus* and Other Staphylococci

**DOI:** 10.3390/vetsci12060568

**Published:** 2025-06-10

**Authors:** Alba Martinez-Laorden, Celia Arraiz-Fernandez, Gonzalo Ibañez-Torija, Elena Gonzalez-Fandos

**Affiliations:** Food Technology Department, CIVA Research Center, University of La Rioja, Madre de Dios 53, 26006 Logrono, La Rioja, Spain

**Keywords:** foodborne, MRSA, antimicrobial resistance, *S. aureus*, *Mammaliicoccus* spp., *Macrococcus* spp.

## Abstract

Nowadays, there is a great concern about the prevalence of multidrug-resistant bacteria in meat, especially *Staphylococcus aureus* resistant to methicillin (MRSA). The aim of this work was to evaluate the microbiological quality of fresh pork meat and the presence and resistance of *S. aureus* and other *Staphylococcus* spp., *Mammaliicoccus* spp., and *Macrococcus* spp. to methicillin and other antibiotics. A total of 39 fresh pork meat samples were analyzed. MRSA was isolated from one sample, being resistant to eight antimicrobial families, including aminoglycosides, β-lactams, lincosamides, macrolides, sulfonamides, tetracycline, and others such as fusidic acid, and mupirocin. Also, other multidrug-resistant strains with resistance to 5 or 6 antimicrobial families were isolated including *Staphylococcus succinus*, *Mammaliicoccus sciuri*, and *Macrococcus caseolyticus*. Additional measures should be taken to control MRSA in pork meat.

## Simple Summary

Nowadays, there is a great concern about *S. aureus* resistant to methicillin (MRSA). This pathogen poses a major challenge to both human and animal health due to the severity of the infections that they can cause. MRSA can be present in pork meat, being the primary reservoir of the porcine nasopharyngeal tract, although cross-contamination during processing can also occur. This study evaluates the presence of this pathogen in pork meat as well as its antimicrobial resistance. The results show that multi-resistant MRSA can be present in meat. Thus, additional measures should be taken to control this pathogen in pork meat.

## 1. Introduction

Pork meat contains a large amount of nutrients that are essential for human development and for preventing deficiencies, making it a key component of the diet [1,2,3]. It is the most widely consumed meat, with annual consumption exceeding 115 million tons, and it is expected to grow by 8% in 2033 [4]. In the European Union, and especially in Spain, the pork sector plays an important role both in terms of production volume and economic value, with Spain being the largest producer of pork in the EU [5].

Fresh meat is a highly perishable food and is susceptible to microbiological contamination [6]. This contamination can occur at various stages of the production chain, from slaughter to retail sale [7]. Microorganisms identified in pork meat and pigs include *Yersinia enterocolitica*, *Escherichia coli*, *S. aureus*, *Enterococcus* spp., *Listeria monocytogenes*, *Campylobacter* spp., *Clostridium* spp., *Salmonella* spp., and *Pseudomonas* spp. [8,9,10,11,12,13,14,15,16,17,18,19,20]. Some of these microorganisms are spoilage organisms, while others are pathogenic. The latter can cause infections in animals and humans and represent a public health risk, particularly when they exhibit resistance to commonly used antibiotic treatments [21,22].

Among the most concerning pathogens in pork meat is *S. aureus*. *S. aureus* and other staphylococci are associated with cross-contamination of pork, with the primary reservoir being the porcine nasopharyngeal tract. Therefore, most contamination events are attributed to the slaughter and carcass-cutting processes [23]. Several studies have reported the presence of *S. aureus* in a wide range of pork meat samples, with prevalence rates ranging from 6% to 100% [9,10,24,25]. A particularly alarming aspect of this pathogen is its resistance to methicillin, which gives rise to methicillin-resistant *S. aureus* (MRSA). These strains pose a major challenge to both human and animal health due to the severity of the infections they can cause, as they produce toxins and enterotoxins capable of inducing food poisoning, as well as urinary, respiratory, and bloodstream infections [25,26,27]. Pigs have been identified as a significant reservoir of MRSA, highlighting the urgent need to implement control measures during the production and processing stages [28]. Similarly, the presence of MRSA strains has been detected in pork meat [10,24,25,29,30,31,32,33]. Likewise, the presence of other methicillin-resistant staphylococci (MRS) has also been reported in pigs [34].

Due to the use of antibiotics in livestock, animals have become one of the main reservoirs of antibiotic-resistant bacteria, such as *Staphylococcus* spp., which can spread to humans through the consumption of contaminated meat, among other routes [35,36]. This poses a significant risk to both human and animal health, as it considerably reduces the effectiveness of antibiotic treatments [37]. In response to this issue, the World Health Organization (WHO), the Food and Agriculture Organization of the United Nations (FAO), and the World Organisation for Animal Health (OIE) promote the “One Health” concept, which advocates for a unified approach to human, animal, and environmental health, with the aim of generating rapid and effective responses to emerging health challenges [38,39].

The microbiological analysis of pork available on the market is essential not only for detecting potential microbiological risks but also for evaluating the effectiveness of hygiene and preservation practices at retail points. In Spain, although strict food safety regulations are in place, microbiological surveillance at the retail level remains a critical point for the prevention of foodborne illnesses [40].

The objective of this study is to characterize the microbiological profile of fresh pork meat, as well as the resistance of *S. aureus* and other *Staphylococcus* spp., *Mammaliicoccus* spp., and *Macrococcus* spp. to methicillin and other antibiotics.

## 2. Materials and Methods

### 2.1. Sampling and Microbiological Evaluation

A total of 39 fresh pork meat samples were obtained from 9 different retail locations in La Rioja, Spain. The samples were collected between January 2020 and January 2021 as part of a preliminary study focused on detecting antibiotics in meat [41]. The number of samples was determined based on consumption data, product availability, and the variety of commercial brands [42]. The samples were ensured to meet optimal storage standards, maintaining a temperature of 4 °C from the time of purchase until arrival at the laboratory. For microbiological analyses, 10 g of pork meat was homogenized using a stomacher (IUL Instruments, Barcelona, Spain) and sterile peptone water (0.1% *w*/*v*) (Oxoid, Basingstoke, Hampshire, UK). The following determinations were carried out: *Enterobacteriaceae* (Oxoid), *Pseudomonas* spp. (Scharlau, Barcelona, Spain), mesophilic bacteria (Scharlau), and staphylococci (Oxoid), as well as the detection of methicillin-resistant *S. aureus* using ChromID MRSA medium (Biomerieux, Marcy l’Etoile, France), as previously described by Martínez-Laorden et al. [43,44].

### 2.2. Isolation and Identification of Strains

Five colonies per medium and sample were selected for strain isolation and identification. The selected colonies were purified using brain–heart infusion broth (Scharlau) and tryptic soy agar (Scharlau) and were then stored at −80 °C. Identification of the purified isolates was performed using MALDI-TOF mass spectrometry (Bruker, Daltonik, Bremen, Germany).

### 2.3. Phenotypic Confirmation of Methicillin-Resistant Micrococcaceae

Phenotypic determination of methicillin-resistant *Staphylococcus* spp., *Mammaliicoccus* spp., and *Macrococcus* spp. was conducted following the guidelines of the Clinical Laboratory Standards Institute using the disc diffusion technique [45]. A strain of *S. aureus* sensitive to methicillin and another resistant to methicillin belonging to the collection of the University of La Rioja were included for quality control.

### 2.4. Phenotypic Evaluation Assay of Staphylococcus spp., Mammaliicoccus spp., and Macrococcus caseolyticus

A phenotypic resistance evaluation assay was performed on *Staphylococcus* spp., *Mammaliicoccus* spp., and *Macrococcus caseolyticus* strains following the disc diffusion method as described by Martínez-Laorden et al. [43,44]. The following antibiotics were used: vancomycin, lincomycin, levofloxacin, gentamicin, ceftaroline, nitrofurantoin, ciprofloxacin, fusidic acid, tedizolid, amikacin, enrofloxacin, kanamycin, linezolid, minocycline, gatifloxacin, erythromycin, ampicillin, chloramphenicol, mupirocin, quinupristin/dalfopristin, trimethoprim, benzylpenicillin, streptomycin, rifampicin, norfloxacin, sulfadiazine, cefoxitin, trimethoprim-sulfamethoxazole, clindamycin, tobramycin, penicillin, doxycycline, tetracycline, and tylosin (Oxoid). A strain was considered multiresistant when it showed resistance to three or more families of antibiotics [43,44].

### 2.5. Statistical Analysis

Statistical analysis was carried out using SPSS software version 28.0.1.1 (IBM SPSS Statistics, Armonk, NY, USA). Tukey’s test for comparison of means was performed using the same program. The level of significance was determined at *p* < 0.05.

## 3. Results

Table 1 shows the microbiological counts obtained from fresh pork meat samples. Mesophilic bacteria showed the highest values, being detected in all samples (mean: 4.93 ± 1.37 log CFU/g). *Pseudomonas* spp. was found in 50% of the samples, with a mean of 3.89 ± 1.17 log CFU/g, while *Enterobacteriaceae* showed a mean of 3.46 ± 0.86 log CFU/g, with 35.9% of the samples exceeding 1 log CFU/g. Finally, staphylococci showed the lowest counts (mean: 2.16 ± 0.74 log CFU/g), although they were present in a considerable number of samples.

A total of 113 strains were isolated from the PCA medium, distributed across various microbial groups as shown in Table 2. The predominant species was *Brochothrix thermosphacta* (26.55%), followed by the genus *Pseudomonas* spp. (22.12%), with *Pseudomnas fragi* (12.39%) and *Pseudomonas lundensis* (7.96%) being the most frequently identified. Within the lactic acid bacteria (LAB) (19.47%), *Carnobacterium divergens* (14.16%) and *Carnobacterium maltaromaticum* (2.65%) were the most notable. The *Micrococcaceae* represented 12.39% of the total. *Enterobacteriaceae* made up 8.85% of the isolates, with *Rahnella insidiosa* (3.54%) and *Hafnia alvei* (2.65%) being the most predominant.

Regarding the *Pseudomonas* genus isolated from the specific agar for this group, a total of 13 different species were identified as can be seen in Table 3. The most prevalent was *Pseudomonas libanensis*, with 32 isolates, representing 41.03% of the total, followed by *Pseudomonas fluorescens* (14.10%) and *Pseudomonas azotoformans* (10.26%). Other species with notable presence included *P. antarctica* (8.97%) and *Pseudomonas extremorientalis* (6.41%). Less common species, such as *Pseudomonas poae*, *Pseudomonas rhodesiae*, *Pseudomonas. tolaasii*, and *Pseudomonas trivialis*, were represented by a single isolate each (1.28%).

Regarding the species identified within the *Enterobacteriaceae* group and other Gram-negative fermentative bacilli isolated from agar MacConkey, shown in Table 3, 12 different species were observed, with *Serratia liquefaciens* being the most prevalent (32.31%), followed by *Rahnella aquatilis* (20%) and *Hafnia alvei* (13.85%). Also, *Ewingella americana* (7.69%), *Buttiaxella warmboldiae* (7.69%), *Klebsiella oxytoca* (4.62%), *Pantoea agglomerans*, (4.62%), and *Buttiaxella gaviniae* (4.62%) were isolated from fresh pork meat. The detection of *Yersinia enterocolitica* is noteworthy, although it occurred at a low frequency (1.54%), due to its relevance as a foodborne pathogen.

Regarding the Gram-positive cocci isolated from mannitol salt agar presented in Table 3, 10 different species were identified, with *Staphylococcus saprophyticus* predominating (38.78%), followed by *Staphylococcus warneri* (12.24%), and *Macrococcus caseolyticus* (12.24%). Other species from the *Staphylococcus* spp. were also detected, such as *Staphylococcus equorum* (10.20%), *Staphylococcus epidermidis* (4.08%), and *Staphylococcus succinus* (4.08%), in addition to members of the *Mammaliicoccus* spp., such as *Mammaliicoccus sciuri* (8.16%), *Mammaliicoccus vitulinus* (6.12%), and *Mammaliicoccus fleurettii* (2.04%).

With the use of ChromID MRSA medium, MRSA was detected in only one sample, and confirmed as *S. aureus* by MALDI-TOF.

Table 4 presents the antimicrobial resistance phenotypic profile of various *Staphylococcus* spp., *Mammaliicoccus* spp., and *Macrococcus caseolyticus* strains isolated from fresh pork meat. The presence of multidrug resistance in several strains is highlighted, defined as resistance to three or more classes of antibiotics. In particular, *S. aureus* isolated from ChromID MRSA medium showed resistance to eight antimicrobial families, including aminoglycosides (amikacin, streptomycin, tobramycin), β-lactams (cefoxitin), lincosamides (clindamycin), macrolides (lincomycin), sulfonamides (sulfadiazine), tetracyclines (tetracycline), and others such as fusidic acid and mupirocin. This strain was also classified as methicillin-resistant (MR).

Other multidrug-resistant strains included *S. succinus*, *M. sciuri*, and *Macrococcus caseolyticus*, showing resistance to 5 or 6 antimicrobial families and also classified as methicillin-resistant.

## 4. Discussion

Several studies have established that mesophilic levels ranging from 6 to 8 log CFU/g are associated with pork in good quality condition, while values exceeding these indicate deteriorating and poor meat quality [46,47]. Based on this parameter, the current study detected a mesophilic mean count of 4.93 ± 1.37 log CFU/g, indicating an appropriate quality state of the analyzed pork according to the mentioned criteria. These results are higher than those found by Yan et al. [48], who described counts of 2.33 ± 0.02 log CFU/g in pork samples purchased in China.

Other authors report mesophilic counts in pork ranging from 2.92 to 5.34 log CFU/g, data like those obtained in our study and positively correlating with the quality criteria mentioned earlier [49,50,51]. In addition, the observed differences may be due to differences in storage conditions and storage time of the samples [52].

In our current study, *B. thermosphacta* and *Pseudomonas* spp. were identified as the predominant group in the microbiota of the analyzed samples. They were followed by lactic acid bacteria (LAB), *Micrococcaceae*, *Enterobacteriaceae*, other Gram-negative bacteria (*Acinetobacter* spp., *Moraxella osloensis*, and *Stenotrophomonas rhizophila*), and other Gram-positive bacteria (*Dermacoccus nishinomiyaensis*). *Pseudomonas* spp. and *B. thermosphacta* have been previously described as the main bacterial agents responsible for meat spoilage, including pork meat [43,44,53]. These findings correspond with those mentioned by other authors, such as Papadopoulou et al. [54], who pointed out *Pseudomonas* spp., *B. thermosphacta*, LAB, and *Enterobacteriaceae* as the main bacterial groups detected in pork meat samples in Greece. It is noteworthy that elevated levels of *B. thermosphacta* could be associated with an unacceptable quality state pork meat, as this bacterium is linked to food spoilage [55].

In relation to *Pseudomonas* spp. counts, in this study, mean values of 3.89 ± 1.17 log CFU/g were observed. Similar values, but somewhat higher have been reported by other authors, who observed *Pseudomonas* spp. counts in pork between 2.99 and 3.56 log CFU/g [56,57].

In this study, the presence of *Pseudomonas* spp. was observed, with isolates identified as *P. libanensis*, *P. fluorescens*, *P. azotoformans*, *P. antarctica*, *P. veronii*, *P. fragi*, *P. synxantha*, *P. marginalis*, *P. rhodesiae*, *P. tolaasii*, *P. poae*, *P. lundensis*, *P. extremorientalis*, and *P. trivialis*. The presence of *Pseudomonas* spp. is associated with food contamination [58,59]. Additionally, other authors have reported the presence of *P. fragi*, *P. libanensis*, *P. synxantha*, *P. poae*, *P. trivialis*, *P. tolaasii*, and *P. fluorescens*, among others, isolated from pork samples in Italy [60], as well as the species *P. aeruginosa*, *P. brenneri*, *P. cedrina*, *P. fulva*, *P. gesardii*, *P. kilonensis*, *P. orientalis*, *P. proteolytica*, *P. putida*, and *P. taetrolens*, which were not identified in the present investigation. Authors such as Dorn-In et al. [61] also identified several species of Pseudomonas spp. in samples of fresh pork meat purchased in Germany.

The Food and Agriculture Organization (FAO) considers, as a meat quality criterion for consumption, that it is suitable if *Enterobacteriaceae* counts are below 3 log CFU/g [62]. The mean of the counts in the present study exceeds this value, 3.46 ± 0.86 log CFU/g, which could indicate the presence of the beginning of meat spoilage. Other studies have reported *Enterobacteriaceae* counts in pork ranging from 1 to 2.61 log CFU/g, values that would meet the mentioned quality criteria [49,50]. In China, Liang et al. [57] observed *Enterobacteriaceae* counts in pork of 3.38 log CFU/g, like those observed in this study. On the other hand, other studies have reported counts of 4.2 ± 0.99 log CFU/g in pork samples purchased in South Africa [63], higher than those found in the present investigation. The presence of *Enterobacteriaceae* has been related to improper handling of pork during processing [48].

In this study, various species of *Enterobacteriaceae* have been identified, with *S. liquefaciens*, *R. aquatilis*, and *H. alvei* standing out as the most prevalent among the isolated *Enterobacteriaceae*. Strains of *Buttiauxella* spp., *P. agglomerans*, *K. oxytoca*, *E. americana*, *Serratia grimesii*, and *Y. enterocolitica* have also been detected. Other researchers have reported the presence of *Buttiauxella* spp., *P. agglomerans*, *Klebsiella pneumoniae*, and *Yersinia massiliensis* in pork samples [60]. The presence of *Y. enterocolitica* is concerning, as it is associated with yersiniosis, an infection that may require hospitalization and, in severe cases, can be fatal for the infected individual [64]. Other studies have reported the presence of *Y. enterocolitica* strains in pork samples from Mexico, China, and Italy [65,66,67].

On the other hand, *E. coli* was not observed among the identified strains, although its presence in fresh pork meat acquired in Germany, China, France, and Italy has been described by other authors [60,61,68,69,70,71].

As for *Staphylococcus* spp. in our study, mean counts of 2.16 ± 0.74 log CFU/g were detected, a higher value than that found by Meng et al. [72] in pork samples in China (1.35 ± 0.33 log CFU/g) and Dorn-In et al. [61] in pork samples in Germany (1.5 log CFU/g).

In the present study, several strains of *Kokuria* spp. (*K. palustris*, *K. rhizophila*, and *K. sasicia*), *Staphylococcus* spp., and the species *M. caseolyticus* have been observed. Other studies also describe the presence of these bacteria in pork samples, coinciding with those found in our study, by detecting the presence of *K. palustris*, *K. rhizophila*, *K. sasicia*, *M. caseolyticus*, and strains of *Staphylococcus* spp. [60,61]. In a study carried out with fresh pork meat in Korea, *Kocuria* spp. as well as *M. caseolyticus* were isolated, coinciding with our results [73].

In the current research, six species of *Staphylococcus* spp. were found (*S. aureus*, *S. epidermidis*, *S. equorum*, *S. saprophyticus*, *S. succinus,* and *S. warneri*), as well as three species of *Mammaliicococcus* spp. (*M. fleuretii*, *M. sciuri*, and *M. vitulinus*), and the species *M. caseolyticus*. Other publications in Germany and Italy have described the identification of several species of Staphylococcus spp. isolated from pork meat, such as *S. aureus*, *S. capitis*, *S. succinus*, *S. equorum*, *S. epidermidis*, *S. warneri*, *S. xylosus*, *S. chromogenes*, *S. haemolyticus*, *S. hominis*, *S. pasteuri*, *S. saprophyticus,* and *S. simulans*, as well as the species *M. caseolyticus* [60,61]. These differences from our study could be due to the possibility of cross-contamination during carcass processing in slaughterhouses [74].

In the present study, phenotypic analysis was performed on 18 *Staphylococcus* spp. isolates, with only one identified as *S. aureus*. This strain was classified as methicillin-resistant and exhibited resistance to 8 different antibiotic families and 14 individual antibiotics. Other authors have reported the identification of 43 *S. aureus* strains out of a total of 99 pork samples in Oklahoma, which showed multi-resistance, with 100% resistance to ampicillin, 94.8% to tetracycline, 88.7% to penicillin, and 83.5% to doxycycline; 40.9% of the strains were classified as methicillin-resistant [29]. These findings contrast with those of the present study, where the *S. aureus* strain analyzed only showed resistance to tetracycline and penicillin among the antibiotics mentioned. 

In a study conducted in Brazil using pork samples, 73 *S. aureus* strains were identified, 54 of which were classified as MRSA. Among these, 36.99% were resistant to tetracycline and 6.85% to vancomycin [32]. In contrast, in our study, all *Staphylococcus* spp. strains analyzed were susceptible to vancomycin. It is noteworthy that *Staphylococcus* spp. strains resistant to erythromycin (33.33%) were detected. Erythromycin is included in “Category B: restricted use in humans” of the Spanish National Action Plan on Antimicrobial Resistance [75]. The acquisition of infections through the consumption of food contaminated with bacteria resistant to antibiotics reserved for human use poses a serious threat to both human and animal health.

In this study, a strain of *S. epidermidis* was isolated that exhibited multidrug resistance to 5 antibiotic families and resistance to 6 antibiotics but it was not methicillin-resistant, while *S. warneri* was found to be resistant to 6 antibiotic families in addition to being methicillin-resistant. Other authors observed two strains of *S. epidermidis* isolated from pork, one resistant to ampicillin, cefoxitin, and gentamicin, and another strain resistant to cefoxitin, erythromycin, mupirocin, and tetracycline, both strains methicillin-resistant and multidrug-resistant against three and four antibiotic families, respectively [76].

Regarding the strains of *Mammaliicococcus* spp. and *M. caseolyticus* analyzed, two strains of *M. caseolyticus* and one of *M. sciuri* showed resistance to multiple drugs, and the strain of *M. sciuri* and one of the strains of *M. caseolyticus* were resistant to methicillin. Some authors have found strains of *Mammaliicococcus* spp. and *M. caseolyticus* isolated from pork resistant to antibiotics, but they do not specify against which ones [77,78]. No further studies describing the antibiotic sensitivity of *Mammaliicococcus* spp. and *M. caseolyticus* strains isolated from pork have been found. *M*. *caseolyticus* species and *Mammaliicococcus* spp. are closely related to *Staphylococcus* spp. and are potential disseminators of antibiotic resistance [34,79,80].

Snyder et al. [81] elucidated an average transfer of MRSA from contaminated pork products to cutting boards, ranging between 39% and 49%, depending on the type of product. Using an innovative dose–response colonization model for *S. aureus* proposed by Schoen et al. [82], the significant threat of the anticipated risk of nasal colonization by MRSA due to the handling of contaminated pork meat at the retail level was revealed. The identification of an MRSA isolate resistant to multiple antibiotics in pork meat samples raises a serious concern in terms of public health.

This finding highlights the importance of addressing and mitigating the spread of resistant strains in meat products not only from a human health perspective, but also from an animal and environmental health perspective, in accordance with the “One Health” paradigm. This approach allows for the implementation of faster and more targeted measures to tackle this issue [35].

In the present work, only phenotypic determination of methicillin-resistant *Micrococcaceae* was carried out. Since no molecular determination was undertaken, further studies are needed.

## 5. Conclusions

This study highlights that fresh pork meat can be a source of *S. aureus* resistant to methicillin (MRSA) and other antimicrobials. Moreover, pork meat can be a source of other multi-resistant strains belonging to the following species: *S. succinus*, *M. sciuri*, and *M. caseolyticus*. The presence of multi-resistant bacteria in fresh pork meat is of special concern. Additional measures should be taken within the framework of the “One Health” approach.

## Figures and Tables

**Table 1:** Microbiological load (log CFU/g) detected in 39 samples of fresh pork meat.

Microbial Group	N ^1^ Counts < 1 log CFU/g	N Counts > 1 Log CFU/g	Minimum Value (log CFU/g)	Maximum Value (log CFU/g)	Mean ± Standard Deviation
Mesophiles	0	39	2.00	7.45	4.93 ± 1.37
Pseudomonas	19	20	2.00	6.15	3.89 ± 1.17
*Enterobacteriaceae*	25	14	1.60	4.49	3.46 ± 0.86
Staphylococci	24	15	1.30	3.92	2.16 ± 0.74

^1^ Number of samples.

**Table 2:** Bacterial species isolated from PCA in fresh pork meat.

Microbial Group (N ^1^; Percentage)	Specie	Number of Strains	Percentage (%)
*Pseudomonas* spp. (25; 22.12%)	*Pseudomonas extremorientalis*	1	0.88
	*Pseudomonas fragi*	14	12.39
	*Pseudomonas libanensis*	1	0.88
	*Pseudomonas lundensis*	9	7.96
*Micrococcaceae* (14; 12.39%)	*Kocuria palustris*	1	0.88
	*Kocuria rhizophila*	6	5.31
	*Kocuria salsicia*	4	3.54
	*Macrococcus caseolyticus*	1	0.88
	*Staphylococcus warneri*	2	1.77
BAL (22; 19.47%)	*Carnobacterium divergens*	16	14.16
	*Carnobacterium maltaromaticum*	3	2.65
	*Lactobacillus* sp.	2	1.77
	*Lactococcus lactis*	1	0.88
*Enterobacteriacecae* (10; 8.85%)	*Citrobacter braakii*	1	0.88
	*Hafnia alvei*	3	2.65
	*Rahnella inusitata*	4	3.54
	*Serratia proteamaculans*	2	1.77
Brochothrix thermosphacta (30; 26.55%)	*Brochothrix thermosphacta*	30	26.55
Other Gram-negative bacteria (9; 7.96%)	*Acinetobacter guillouiae*	2	1.77
	*Acinetobacter harbinensis*	4	3.54
	*Acinetobacter lactucae*	1	0.88
	*Stenotrophomonas rhizophila*	1	0.88
	*Moraxella osloensis*	1	0.88
Other Gram-positive bacteria (3; 2.65%)	*Dermacoccus nishinomiyaensis*	3	2.65

^1^ Number of strains isolated.

**Table 3:** Bacterial species isolated from specific media in fresh pork meat.

Media (N ^1^)	Species	Number of Strains	Percentage (%)
Chromogenic Pseudomonas Agar (78)	*Pseudomonas antarctica*	7	8.97
	*Pseudomonas azotoformans*	8	10.26
	*Pseudomonas extremorientalis*	5	6.41
	*Pseudomonas fluorescens*	11	14.10
	*Pseudomonas fragi*	2	2.56
	*Pseudomonas libanensis*	32	41.03
	*Pseudomonas marginalis*	2	2.56
	*Pseudomonas poae*	1	1.28
	*Pseudomonas rhodesiae*	1	1.28
	*Pseudomonas synxantha*	3	3.85
	*Pseudomonas tolaasii*	1	1.28
	*Pseudomonas trivialis*	1	1.28
	*Pseudomonas veronii*	4	5.13
MacConkey Agar (65)	*Buttiauxella gaviniae*	3	4.62
	*Buttiauxella warmboldiae*	5	7.69
	*Ewingella americana*	5	7.69
	*Hafnia alvei*	9	13.85
	*Klebsiella oxytoca*	3	4.62
	*Pantoea agglomerans*	3	4.62
	*Rahnella aquatilis*	13	20.00
	*Serratia grimesii*	2	3.08
	*Serratia liquefaciens*	21	32.31
	*Yersinia enterocolitica*	1	1.54
Mannitol Salt Agar (49)	*Kocuria uropygioeca*	1	2.04
	*Macrococcus caseolyticus*	6	12.24
	*Mammaliicoccus fleurettii*	1	2.04
	*Mammaliicoccus sciuri*	4	8.16
	*Mammaliicoccus vitulinus*	3	6.12
	*Staphylococcus epidermidis*	2	4.08
	*Staphylococcus equorum*	5	10.20
	*Staphylococcus saprophyticus*	19	38.78
	*Staphylococcus succinus*	2	4.08
	*Staphylococcus warneri*	6	12.24

^1^ Number of strains isolated.

**Table 4:** Antimicrobial resistance phenotypic profile of *Staphylococcus* spp., *Mammaliicoccus* spp., and *Macrococcus caseolyticus* strains isolated from fresh pork meat.

Identification	Phenotypic Pattern of Resistance ^1^	Multirresist ^2^	MR ^3^
*Staphylococcus aureus*	AK-S-K-TOB-FOX-CMN-MY-PNG-P-SUZ-MH-TE-FAD-PUM ^C^	Yes (8) ^5^	Yes
*Staphylococcus epidermidis*	TOB-ERY-P-SUZ-DO-TE ^B^	Yes (5)	No
	- ^4B^	No	No
*Staphylococcus equorum*	ERY-W ^B^	No	No
	MY-ERY ^B^	No	No
	- ^B^	No	No
*Staphylococcus saprophyticus*	ERY-FAD ^B^	No	No
	PNG-P-DO-TE ^B^	No	No
	- ^B^	No	No
	- ^B^	No	No
	- ^B^	No	No
	DO-TE ^B^	No	No
*Staphylococcus succinus*	- ^B^	No	No
	- ^B^	No	No
*Staphylococcus warneri*	S-TOB-FOX-P-SUZ-FAD-PUM ^B^	Yes (6)	Yes
	ERY ^A^	No	No
	MY-P ^B^	No	No
	ERY ^A^	No	No
*Mammaliicoccus fleurettii*	MY ^B^	No	No
*Mammaliicoccus sciuri*	MY-FAD ^B^	No	No
	FOX-MY-P-PUM ^B^	Yes (4) ^5^	Yes
*Mammaliicoccus vitulinus*	- ^B^	No	No
	MY ^B^	No	No
*Macrococcus caseolyticus*	CMN-MY-ERY-TE ^B^	Yes (3) ^4^	No
	MY-ERY ^B^	No	No
	MY-ERY ^A^	No	No
	AK-FOX-CPT-MY-P-PUM ^B^	Yes (5)	Yes

^1^ The letters indicate the isolation medium. ^A^ Agar Plate Count. ^B^ Agar Manitol Sal. ^C^ ChromID MRSA. ^2^ Multidrug resistance. ^3^ Methicillin-resistant. ^4^ Susceptible to all the antibiotics tested. ^5^ Number of antibiotic families to which the strain is resistant. AK: amikacin, S: streptomycin, K: kanamycin, TOB: tobramycin, FOX: cefoxitin, CMN: clindamycin, MY: lincomycin, ERY: erythromycin, P: penicillin, SUZ: sulfadiazine, DO: doxycycline, MH: minocycline, TE: tetracycline, FAD: fusidic acid, PUM: mupirocin, W: trimethoprim, PNG: benzylpenicillin.

## Data Availability

Data will be made available on request.

## Abbreviations

The following abbreviations are used in this manuscript:

MRSA	*Staphylococcus aureus* resistant to methicillin
MRS	Methicillin-resistant staphylococci
MR	Methicillin-resistant
